# Curcumin Loaded Polymeric vs. Lipid Nanoparticles: Antioxidant Effect on Normal and Hypoxic Olfactory Ensheathing Cells

**DOI:** 10.3390/nano11010159

**Published:** 2021-01-10

**Authors:** Angela Bonaccorso, Rosalia Pellitteri, Barbara Ruozi, Carmelo Puglia, Debora Santonocito, Rosario Pignatello, Teresa Musumeci

**Affiliations:** 1 Department of Drug Sciences, University of Catania, V.le Andrea Doria, 6, 95125 Catania, Italy; abonaccorso@unict.it (A.B.); capuglia@unict.it (C.P.); debora.santonocito@outlook.it (D.S.); r.pignatello@unict.it (R.P.); 2 Institute for Biomedical Research and Innovation, National Research Council, Via Paolo Gaifami 18, 95126 Catania, Italy; 3 Department of Life Sciences, University of Modena and Reggio Emilia, 41124 Modena, Italy; barbara.ruozi@unimore.it

**Keywords:** intranasal delivery, nanomedicine, olfactory ensheathing cells, curcumin, PLGA

## Abstract

Background: Curcumin (Cur) shows anti-inflammatory and antioxidant effects on central nervous system diseases. The aim of this study was to develop Cur-loaded polymeric and lipid nanoparticles for intranasal delivery to enhance its stability and increase antioxidant effect on olfactory ensheathing cells (OECs). Methods: The nanosuspensions were subjected to physico-chemical and technological evaluation through photon correlation spectroscopy (PCS), differential scanning calorimetry (DSC) and UV-spectrophotometry. The cytotoxicity studies of nanosuspensions were carried out on OECs. A viability test was performed after 24 h of exposure of OECs to unloaded and curcumin-loaded nanosuspensions. The potential protective effect of Cur was assessed on hypoxic OECs cells. Uptake studies were performed on the same cell cultures. Thermal analysis was performed to evaluate potential interaction of Cur with a 1,2-Dimyristoyl-*sn*-glycero-3-phosphocholine (DMPC) biomembrane model. Results: PCS analysis indicated that lipid and polymeric nanosuspensions showed a mean size of 127.10 and 338.20 nm, respectively, high homogeneity and negative zeta potential. Incorporation of Cur into both nanocarriers increased drug stability up to 135 days in cryoprotected freeze-dried nanosuspensions. Cell viability was improved when hypoxic OECs were treated with Cur-loaded polymeric and lipid nanosuspensions compared with the control. Conclusions: Both nanocarriers could improve the stability of Cur as demonstrated by technological studies. Biological studies revealed that both nanocarriers could be used to deliver Cur by intranasal administration for brain targeting.

## 1. Introduction

The nasal route has been increasingly applied in attempts to deliver drugs for local treatment and even for systemic and brain delivery [1]. Intranasal administration offers many advantages: firstly, it is minimally invasive promoting medication adherence in patients; it is painless; and it bypasses the gastrointestinal and hepatic first-pass metabolism that can inactivate a substantial fraction of the administered drug [2,3]. However, this route presents some disadvantages due to the site of administration, in fact, when the drug molecule enters into the nasal cavity, it experiences mucociliary clearance in the vestibular region which restricts foreign particles and the potential local drug enzymatic degradation. Moreover, it is suitable only for potent drugs since a limited volume (100–200 µL) can be sprayed into the nasal cavity [4,5]. On the other hand, nanomedicine, over the last two decades, has been growing quickly and it is used in disease diagnosis and treatment to overcome some pharmacokinetic limits [6]. The combination of nanomedicine and intranasal route can represents a successful strategy to achieve brain targeting bypassing the blood brain barrier (BBB), which is impermeable to nearly 100% of macromolecular drugs and 98% of small molecules and to overcome some drug drawbacks [7,8]. Currently, the growing interest of nose to brain delivery is often associated to the treatment of neurological disorders such as Alzheimer’s disease (AD) because conventional drug delivery methods fail to efficiently deliver pharmaceutical agents to the central nervous system (CNS). AD is a widespread chronic and progressive age-related neurodegenerative disorder, characterized by accumulation of β-amyloid (Aβ), senile plaques, neurofibrillary tangles (NFTs), affects cognitive and memory and induces physical impairment and death [9]. Natural products such as curcumin (Cur) may exert a beneficial role in the management of AD as revealed by in vitro studies that have shown that Cur inhibits Aβ aggregation and Aβ induced inflammation [10]. Furthermore, Cur has been found to improve memory and cognitive deficits in rats [11].

However, Cur pharmacological application has been impeded by its low bioavailability, pH-instability and insolubility in water [12] preventing therapeutic use [13].

Specifically, Cur (1,7-bis (4-hydroxy-3-methoxyphenyl)-1,6-heptadiene-3,5-dione), is a polyphenolic substance, with bright yellow color, obtained from the rhizome of *Curcuma longa* L.

As reported by Nelson et al. (2017), from 1995 to the present, according to the NIH RePORTER database, 30 federal funds exceeding $150 million have been awarded for projects that were linked to the biomedical exploration of Cur [14]. As mentioned previously, the scientific interest is due to several biological activities that are associated with this drug, such as anti-inflammatory, antiproliferative, antioxidant [15], chemo preventive effects [16,17] and its implication in the treatment of neurological disorders [18]. In order to improve Cur stability, it is often formulated in carriers designed to make it suitable for therapeutic applications [19]. These strategies for improving the bioavailability of Cur include the use of solid dispersions, copolymeric micelles, polymeric nanoparticles and microparticles, lipid nanoparticles, liposomes, microemulsions and nanocrystals [5,20,21,22].

In this work, we focused our attention on two different types of nanoparticles (NPs) formulations based on polymeric and lipid components with safe ingredients, respectively, produced using the nanoprecipitation method and a modification of the solvent diffusion technique.

Polymeric NPs can be obtained through different methods and materials. Researchers often focused their attention on biocompatible and biodegradable polymers such as poly(d,l-lactic-co-glycolic acid) (PLGA) that exhibits tunable mechanical properties and it is a Food and Drug Administration (FDA) approved biopolymer [23,24]. On the other hand, solid lipid nanoparticles (SLN) are composed of a lipid core that is solid at room temperature and stabilized with one or a mixture of surfactants [25]. The investigation of Cur for AD has received great attention; in fact, more than 700 published articles can be found in the Pubmed database. A detailed research in the same database revealed that either PLGA NPs, SLN or both have been investigated extensively with regard to Cur encapsulation (181 results with keywords: “curcumin and PLGA nanoparticles”; 43 results with keywords: “curcumin and SLN”; accessed: October 2020); few results were found regarding Alzheimer’s disease, curcumin and both type of NPs (0 results were found with “Alzheimer”, “curcumin” and “SLN” and only 15 results were found with “Alzheimer” together with “curcumin” and “PLGA nanoparticles” accessed: October 2020).

In this regard, we have investigated this aspect and particularly, based on recent scientific findings that indicated that the olfactory nerve undergoes morphological and signals transmission alterations correlated with the development of dementia, we have explored the effect of Cur delivered into polymeric and lipid NPs on normal and hypoxic olfactory ensheathing cells (OECs) [26].

It is known that hypoxia is one of the secondary processes following nerve injury, together with ischemia, axonal loss, cell death, excitotoxicity and inflammation [27]. OECs represent a characteristic glial cell type from the olfactory system showing phenotypic properties with both Schwann cells (SCs) and astrocytes [28]. They are a source of growth factors (GFs), such as glial cell line-derived neurotrophic factor (GDNF), basic fibroblast growth factor (bFGF), brain-derived nerve growth factor (BDNF), nerve growth factor (NGF), and adhesion molecules [29], that allow them to promote axonal regeneration [30,31,32], remyelination [33,34,35] and functional recovery in spinal cord injury (SCI) [36].

Hypoxia is involved in the accumulation of amyloid β (Aβ), hyperphosphorylation of tau, dysfunction of blood–brain barrier (BBB) and degeneration of neurons. The link between the damaging effects of hypoxia and the neurodegeneration of AD has been demonstrated. In fact, hypoxia may disturb the metabolic balance of Aβ, increasing its production and decreasing its degradation [37].

Considering this, the aim of our study was to increase the physico-chemical stability of Cur by encapsulation in two different nanocarrier systems with a polymeric and lipid nature (for convenience abbreviated as PNP and SLN, respectively) and to investigate the effect of Cur loaded in both PNP and SLN in normal and hypoxic OECs evaluating cell viability through MTT test. Cellular uptake of free drug and loaded in PNP and SLN was assessed in normal OECs. Finally, prediction of drug-cellular interaction was performed with a biological membrane model by differential scanning calorimetry (DSC).

## 2. Materials and Methods

### 2.1. Materials

Poly(d,l-lactide-co-glycolide) (PLGA, 719927-5G), polysorbate (Tween) 80, curcumin, D-glucose and hydroxypropyl methyl cellulose (HPMC K15M), acetonitrile and chloroform were purchased from Merk GmbH (Darmstadt, Germany). Acetone was a product from VWR Chemicals, PROLABO (Haasrode, Belgium). Ethanol was purchased from J.T. Baker (Deventer, Holland). Lecinol S-10, hydrogenated lecithin, was obtained from Nikko Chemical (Italy) and Pluronic F68 (poloxamer 188; Lutrol F68) were provided by BASF ChemTrade GmbH (Burgbernheim, Germany). Softisan 100 (Hydrogenated Coco-Glycerides) was a gift from Sasol (Witten, Germany). Furthermore, 1,2-Dimyristoyl-*sn*-glycero-3-phosphocholine (DMPC) was a gift from Corden Pharma International Gmbh (Plankstadt, Germany).

### 2.2. Preparation of Curcumin-Loaded and Unloaded Polimeric Nanoparticles

PNP were prepared by nanoprecipitation method. PLGA (90 mg) was dissolved in acetone (24 mL). The organic phase was dropped into 48 mL of a water/ethanol solution (1:1, *v*/*v*) containing 0.1% (*w*/*v*) Tween 80, under magnetic stirring, obtaining a milky colloidal suspension. The organic solvent was then evaporated off under high vacuum at 40 °C using rotavapor Buchi RE-III. Cur-loaded PNP (Cur-PNP) were obtained by adding Cur (1%, *w*/*w* with respect to the polymer) to the organic phase. The colloidal suspension obtained was subjected to photon correlation spectroscopy (PCS) analysis and subsequently purified by centrifugation at 12,000× *g* for 1 h at 8 °C, using an SL 16R Centrifuge ThermoFisher Scientific.

### 2.3. Preparation of Cur-Loaded and Unloaded SLN Nanoparticles

Blank and Cur-loaded SLN (Cur-SLN) were prepared by using a modification of the solvent diffusion technique [38]. Briefly, Cur (0.02 g, 0.01% *w*/*v*) was dissolved in 4 mL of ethanol and Softisan 100 (0.02 g, 0.01% *w*/*v*) heated to 45 °C approximately. At the same time, an aqueous phase was prepared by dissolving HPMC K15M (0.2 g, 0.1% *w*/*v*), soy lecithin (0.2 g, 0.1% *w*/*v*) and Pluronic F68 (0.2 g, 0.1% *w*/*v*) in 20 mL bidistilled water heated at the same temperature. The organic solution was then injected into the acqueous phase under mechanical stirring at 800 rpm for few minutes. The obtained pre-emulsion was ultrasonified using a UP 400 S (Ultra-schallprozessor, Dr. Hielscher GmbH, Germany) maintaining the temperature at at least 5 °C above the lipid melting point for 10 min. Finally, SLNs were formed in cold water (175 mL) under high-speed homogenization (Ultra-Turrax T25, IKA-Werke GmbH & Co. Kg, Staufen, Germany) at room temperature for 10 min. The suspension was then stored at 4 °C for further characterization.

### 2.4. Particle Size Distribution and Zeta Potential Measurements

PNP and SLN mean size, polydispersity index (PDI) and zeta potential (ZP) were determined 1 h after sample preparation by PCS (Zetasizer Nano S90; Malvern Instruments, Malvern, UK). The experiments were carried out at a detection angle of 90°, at 25 °C with a 4 mW laser operating at 633 nm as light source.

Each value was measured in triplicate. The results are shown as the mean ± standard deviation (SD).

### 2.5. Entrapment Efficiency

The amount of free Cur in the PNP was calculated to determine the entrapment efficiency (EE%). PNP were centrifugated at 12,000 rpm for 1 h at 8 °C using a Thermo Scientific SL16R centrifuge (ThermoFisher Scientific, Waltham, MA, USA) to eliminate unentrapped drug in the supernatant. The amount of Cur in the supernatant was determined spectrophotometrically using a UV-Vis 1601 spectrophotometer (Shimadzu Italia, Milan, Italy) at a wavelength of 470 nm.

The calibration curve for the quantitative evaluation of Cur was linear in the following range: 1.61–25.80 µg/mL (R^2^ = 0.9997); the EE% was calculated using the following equation:

$$Entrapment\;efficiency\;\%=\frac{W\;total-W\;free}{W\;total}\;\times 100$$

where *W total* and *W free* are the weight of Cur initially added in the formulation and the weight of free Cur in the supernatant, respectively.

The percentage of Cur encapsulated into SLN was determined by filtration using a Pellicon XL tangential ultrafiltration system (Millipore, Milan, Italy) equipped with a polyethersulfone Biomax 1000 membrane (molecular weight cut off = 1,000,000). An amount of lyophilized Cur-SLN was solubilized in dichloromethane and the Cur content was measured by UV spectrophotometry at 425 nm (Lambda 52, PerkinElmer, Waltham, MA, USA). Calibration curves for the validated UV assays of drug were run on six solutions in the concentration range 10–100 μg/mL (*R*^2^ > 0.99).

### 2.6. Differential Scanning Calorimetry (DSC) Analysis

DSC scans of raw materials, Cur as well as freeze-dried empty and Cur-loaded PNP and SLN were performed on a Mettler DSC 12E equipped with a Haake thermocryostate model D8-G. A Mettler TA89E and FP89 system software was used for the data acquisition. Indium was used to calibrate the instrument. The reference was an empty aluminum pan. Each sample was analyzed in triplicate at a scan speed of 5 °C/min in a 25–200 °C temperature range.

### 2.7. Morphological Analysis: Scanning Electron Microscopy (SEM) and Transmission Electron Microscopy (TEM)

Cur-PNP morphology was determined using a field emission scanning electron microscope (FE-SEM) (Sigma 300, Calr Zeiss Microscopy GmbH, Jena, Germany). Samples were mounted on a metal stub with double-sided adhesive tape and then sputtered in a vacuum with a chromium layer (Q150T, Quorum Technologies Ltd., Lewes, United Kingdom). Cur-SLN morphology was determined using a transmission electron microscope (JEOL JEM-101).

### 2.8. In Vitro Studies

#### 2.8.1. OECs Cultures

Experimental procedures were carried out according to the Italian Guidelines for Animal Care (D.Lgs 26/2014), and the European Communities Council Directives (2010/63/EU), and were approved by the ethics committee of Catania University (Authorization no. 174/2017-PR). All efforts were made to minimize animal suffering and to reduce the number of animals used. OECs were isolated from 2-d old mouse pup (P2) olfactory bulbs, as reported by [18]. Briefly, the bulbs were removed and digested in medium essential medium-H (MEM-H, Sigma) containing collagenase and trypsin. Trypsinization was stopped by adding Dulbecco’s modified Eagle’s medium (DMEM, Sigma) supplemented with 10% fetal bovine serum (FBS, Sigma). Cells were resuspended and plated in flasks fed with fresh complete medium DMEM/FBS, containing penicillin (50 U/mL) and streptomycin (50 μg/mL). After 24 h cytosine arabinoside, an antimitotic agent was added in order to reduce the number of dividing fibroblasts. OECs were further purified by transferring cells from one flask to a new one. In our experiments, the purity of OECs was assessed through immunocytochemical procedures with p75 and S-100, specific OEC marker. The percentage of S-100/p75 positive cells was about 85–90% (data not shown). Cells were plated on 25 cm^2^ flasks and incubated at 37 °C in a humidified 5% CO_2_–95% air mixture in DMEM/FBS supplemented with bovine pituitary extract and fed twice a week.

#### 2.8.2. Hypoxic Condition

When cells were perfectly attached, the coverslips were upturned in order to obtain hypoxic condition. This procedure reduces oxygen concentration in the cellular environment between the coverslips containing cells and the bottom of multiwells, as reported in our previous papers [18,29,30].

#### 2.8.3. Treatment of Cells

Cur (30 mM; Sigma) was prepared in dimethyl sulfoxide (DMSO); after 24 h post-seeding, six groups (three in normal condition and three in hypoxic condition) were established: (1) cultures with Cur alone; (2) cultures with NPs (PNP and SLN) alone; and (3) cultures with the combination of Cur/NPs at different concentrations (0.1, 0.5, 5 μM Cur diluted in culture medium).

Control cultures (CTR) were grown in DMEM/FBS with the addition of DMSO (5 μL/mL) with no treatment.

#### 2.8.4. Cellular Viability

After 24 h, both in normal and hypoxic conditions, cellular viability with and without NPs, was evaluated by the 3-(4,5-dimethylthiazol-2-yl)-2,5-diphenyl) tetrazolium bromide (MTT, Sigma) reduction assay, a quantitative colorimetric method [39]. Briefly, MTT was added to each multiwell with a final concentration of 1.0 mg/mL and placed in an incubator for 2 h. Media were gently removed and MTT solvent (acid-isopropanol/SDS) was added, then cells were placed on an orbital shaker for 10 min. The absorbance was read by a multisKan reader at 570 nm. Results were expressed as the percentage MTT reduction of control cells.

#### 2.8.5. In Vitro Cellular Uptake Studies

Some OEC cultures were used for uptake studies: Cur-PNP and Cur-SLN at different concentrations (0.1, 0.5 and 5.0 µM) were added to DMEM/FBS medium, while some coverslips were grown with free cur at the same concentration used for both PNP and SLN, as the controls (CTRL), all cells were incubated for 24 h at 37 °C. After incubation, OECs both with free curcumin and with NPs were fixed by exposing them to 4% paraformaldehyde in 0.1 M phosphate-buffered saline (PBS) for 30 min and images were analyzed with a Zeiss fluorescence microscope and captured with an Axiovision Imaging System.

### 2.9. Statistical Analysis

Statistical analysis was performed using Prism 8 (GraphPad Software, Inc., La Jolla, CA, USA) using a one-way analysis of variance (ANOVA) followed by a post hoc Dunnett’s test for the in vitro studies. Significance was defined as *p <* 0.0001.

### 2.10. Biomembrane Models Preparation

Liposomes used as biomembrane models were prepared using the thin layer evaporation (TLE) method. Briefly, DMPC (10 mg) was dissolved in chloroform in a Pyrex glass test-tube. The organic solvent was removed at 30 ± 0.1 °C on nitrogen stream rotavapor (Rotavapor-M Buchi HB-140, Flawil, Switzerland) until the lipids were dried and distributed as a thin film on the wall of the tube. Any possible trace of organic solvent was eliminated by 24 h of storage at 40.0 ± 0.1 °C under high vacuum (Buchi T-50, Flawil, Switzerland). The films were hydrated by adding 400 µL of isotonic PBS (pH 7.4). The tube was alternatively vortexed (Heidolph REAX 2000, Schwabach, Germany) and twice warmed in a water bath at 40 °C for 3 min twice. The temperature was kept higher than that of DMPC gel-liquid crystal phase transition (24 °C) to allow full hydration of the phospholipid. To evaluate the interaction of the drug with liposomes, 5 µM Cur was co-dissolved with DMPC in chloroform.

Samples were subjected to DSC analysis using the same instrument described in Section 2.6. Briefly, the measurements were performed sealing liposomes (as control sample) and liposomes containing Cur in an aluminum pan and 100 µL of isotonic PBS (pH 7.4) was used as reference. Samples were subjected to a heating cycle under a temperature range of 5–55 °C at a scanning rate of 2 °C/min and to two cooling cycles at a scanning rate of 5 °C/min. The main thermotropic parameters (main transition temperature T_m_ and enthalpy changes (∆H)) were calculated.

### 2.11. Lyophilization of Polymeric and Lipid Nanosuspension

Accurately, 5% *w*/*v* glucose as cryoprotectant was added into both nanosuspensions before deep freezing. The nanosuspensions were frozen in a round-bottom flask, using a deep freezer at a temperature below −20 °C for 24 h. The samples were then freeze-dried using an Edwards Modulyo freeze-dryer (Thermo, Waltham, MA, USA) for 24 h at 2 mbar to produce the dry powder. The resulting lyophilized samples were resuspended in distilled water and subjected to PCS analysis.

Freeze-dried Cur-PNP and Cur-SLN were subjected to stability studies as reported in Section 2.13.

### 2.12. Degradation of Free Cur in Phosphate Buffers

The stock solution was prepared by dissolving Cur in ethanol (28 mg/mL). Varying aqueous solution pH values were obtained by mixing Cur stock solution with phosphate buffers (pH 5.8 or pH 7.4). The sample solutions were poured into amber glass bottles. The bottles were then stored at room temperature and in a thermostatically controlled oven set at 37 °C. Sample solutions were taken at predetermined time intervals (0–6 h). The remaining amount of Cur was determined spectrophotometrically after appropriate dilution with ethanol using a UV-Vis 1601 spectrophotometer (Shimadzu Italia, Milan, Italy) at a wavelength of 467 nm (*n* = 3).

### 2.13. Stability Studies

Freeze-dried Cur-PNP and Cur-SLN were stored at room temperature (25.0 ± 1.0 °C/60% relative humidity (RH) ± 5% RH) for a period of 5 months; at specific time intervals, Cur-PNP and Cur-SLN were dissolved in 2 mL of acetonitrile and 4 mL of dichloromethane, respectively, and measured by spectrophotometry (UV-VIS 1601 spectrophotometer, Shimadzu Italia, Milan, Italy) at Cur ʎ max (416 and 467 nm). Calibration curves for the quantitative evaluation of the drug were linear in the following ranges: (i) 10.55–0.844 µg/mL of Cur (R^2^ = 0.9923) for analyses in acetonitrile and (ii) 88.80–5.92 µg/mL of Cur (R^2^ = 0.9984) in dichloromethane. The entrapment of Cur in both lyophilized nanosystems was expressed as % of drug quantity compared to the initial EE%.

## 3. Results

### 3.1. Characterization of Cur-Loaded PNP and SLN

As previously detailed, Cur-SLN were prepared with Softisan 100 (hydrogenated coco-glycerides) and Pluronic F68 (Poloxamer 188) as solid lipid and surfactant, respectively. The modified solvent diffusion technique seemed to be a simple and fast method to prepare SLN. Moreover, Softisan is characterized by a low melting point (35 °C) that allows avoiding of thermal stress conditions which may impair Cur. Cur-SLN showed a mean particle size of about 127.1 nm with respect to blank suspension (112.9 nm).

Moreover, SLN showed a good homogeneity value (0.233 Cur-SLN and 0.194 blank SLN) as represented by PDI values. As revealed by ZP values, both empty and loaded SLN exhibited a net negative charge (−28.6 mV Cur-SLN compared to −32.8 mV for blank SLN, respectively) predicting physical colloidal stability. The SLN formulation showed a very high EE% (~90%).

In the case of empty PNP, we found similar diameters compared to SLN, and a significative size increase after drug loading (Table 1). This finding could confirm the large encapsulation of Cur in the polymeric matrix (PDI 0.145) and a partial drug distribution on particles’ surfaces as revealed by the difference in the ZP values between empty and loaded PNP. The negative ZP is attributed to PLGA due to the carboxyl groups present in its structure [40].

In contrast to SLN, the PNP formulation was found to have EE ~78%. This result confirms the presence of some Cur on NP surface rather than within the polymeric matrix. It is demonstrated by the variation in ZP values between empty and loaded PNP, unlike SLN with higher EE% with surface charge remaining almost unchanged after Cur encapsulation.

This may be due to the increase in the dispersion of the drug in the lipid and also to the presence and the different concentration of the surfactant used in the polymeric vs. lipid formulation. Accordingly, it has been demonstrated that the type and the concentration of surfactant may affect drug encapsulation and that the EE of various SLN stabilized with different nonionic surfactants, decreasing in the order of Poloxamer 188 > Tween 80 > Span 20 [41]. This result was in agreement with the results obtained by Abdelbary et al., formulations containing Span 20 as surfactants showed lower EE compared to the other surfactants; this could be due to the lower hydrophilic-lipophilic balance (HLB) value of Span 20 [42].

In our case, the factors governing the different EE between polymeric and lipid nanosuspensions is the NPs itself, being constituted by different components and prepared by different procedures, and even the presence of two distinct surfactants (Tween 80 and Poloxamer 188, respectively) that according to the authors can affect this parameter [42].

**Figure 1:**
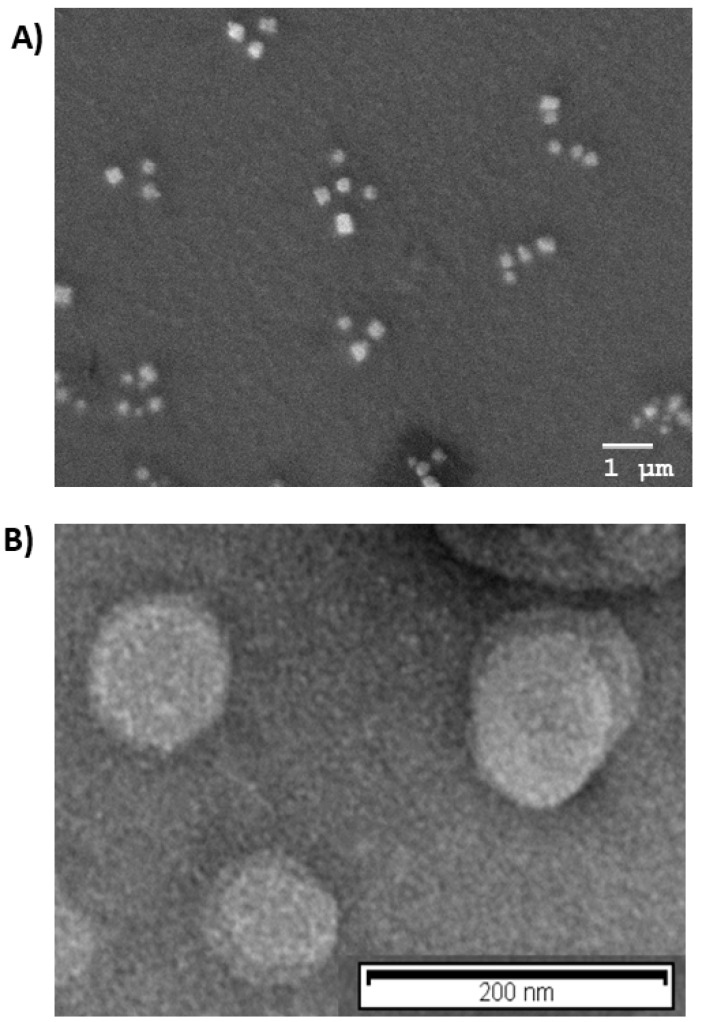
Morphological analysis: (**A**) Scanning electron microscopy (SEM) image of Cur-PNP; (**B**) transmission emission microscopy (TEM) image of Cur-SLN.

Morphological analysis was in agreement with PCS results, as shown in Figure 1A,B, Cur-PNP and Cur-SLN presented spherical shapes; no particle aggregation was detected, confirming that both colloidal systems were homogeneous.

**Figure 2:**
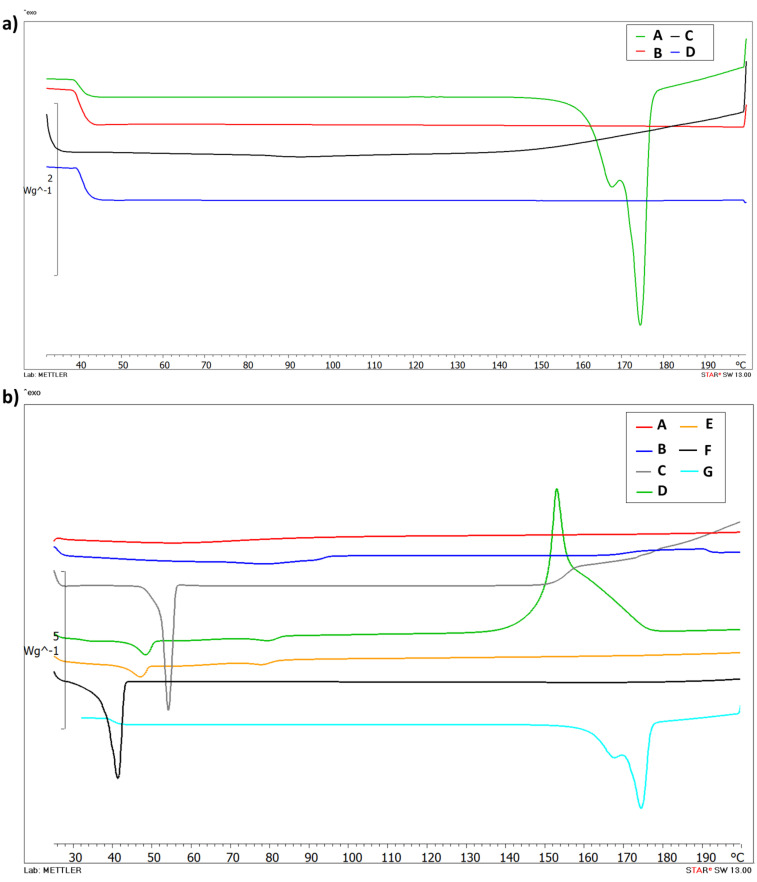
Differential scanning calorimetry (DSC) thermograms of PNP (**a**): Cur (A), PLGA (B), unloaded PNP (C), Cur-PNP (D) and SLN; (**b**): HPMC (A), soy lecithin (B), Lutrol F68 (C), unloaded SLN (D), Cur- SLN (E), Softisan (F) and Cur (G).

DSC analysis was performed to evaluate the thermotropic behavior of Cur-loaded PNP and SLN. Figure 2a shows the thermogram of free Cur (A), PLGA (B), empty PNP (C) and Cur-PNP (D). Cur (A) presents a typical melting endotherm with onset temperature at 169.88 °C indicating its crystalline nature. The absence of melting peak in curves B and C was due to PLGA which is amorphous in nature and showed its typical glass transition at 37 °C [7,43]. Thermal analysis confirmed the encapsulation of Cur in PNP as demonstrated by the absence of Cur peak in the thermogram D that corresponds to Cur-PNP. In Figure 2b the DSC curves of SLN counterpart are shown. Curve F shows the endothermic peak of Softisan with onset temperature at 37.96 °C, while, Lutrol F68 thermogram shows an onset temperature of 51.87 °C (C). Both peaks suffer a slight shift in empty and loaded SLN at 44.49 °C and 73.91 °C, respectively, in the unloaded SLN and at 42.73 °C and 72.89 °C in Cur-SLN.

As suggested by Stella et al. [44] and Carbone et al. [45], lipid melting point variation in SLN colloidal systems compared to the raw material can be due to the formation of the colloidal particles, their dimensions, their high surface-to-volume ratio and to the presence of surfactants, which could also have an influence on this phenomenon.

The thermogram of unloaded SLN (D) shows the exothermic peak at 153.61 °C and it can be related to the degradation process. We observed the disappearance of the exothermic peak in Cur-SLN (E) due to the incorporation of the drug in the lipid matrix. In fact, as demonstrated by Rodenak-Kladniew et al., the incorporation of the drug into the lipid matrix produced some effects on the thermotropic behavior [46]. It could be explained considering the dissolution of the drug in the lipid during the encapsulation process. The changes in lipid matrix after drug loading could suggest modifications of the polymorphic state of the lipid from crystalline to amorphous, which may enhance the drug accommodation associated with high drug encapsulation [46].

The DSC curve E, corresponding to Cur-SLN, did not display any signal related to a Cur endothermic peak. Thus, it can be deduced that the drug incorporated into the polymeric and lipid NPs was in an amorphous or disordered-crystalline state. The change in the thermo-analytical profile of Cur can be attributed to drug dissolution in the lipid and polymeric system.

### 3.2. Cell Viability Evaluation

The OECs are a unique class of glial cells that envelop bundles of olfactory axons, both peripherally in the olfactory nerve and within the olfactory nerve layer of the olfactory bulb [47]. It has been demonstrated that OECs secrete large numbers of neurotrophic factors and promote the migration and survival of neurons [48]. As reported in Velasquez et al., Cur can exert different cellular responses depending on the doses used while activating the same kinases pathways [49,50]. In particular, at high concentrations (10–50 mM) Cur induced apoptosis and autophagy in different cancer cells by activation of extracellular signal-related (ERK) and p38 MAP kinases [51]. However, in neural progenitor cells, high doses of Cur (20–50 mM) are cytotoxic whereas low doses (0.1–0.5 mM) stimulate cell proliferation [52]. Taking into account these considerations, we examined the effect of Cur-loaded PNP and SLN on normal and hypoxic OECs.

**Figure 3:**
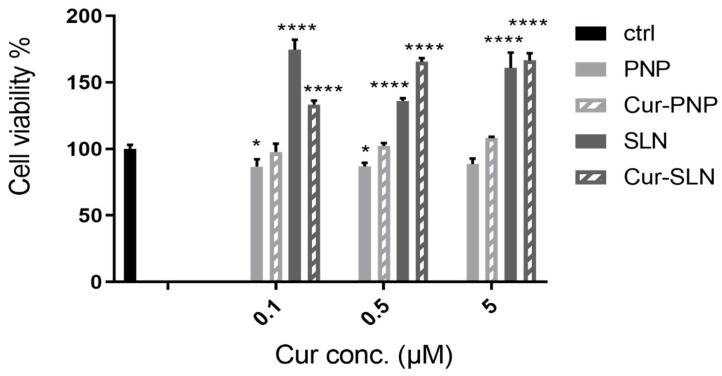
Cell viability after exposure for 24 h of normal olfactory ensheathing cells (OECs) to Cur-PNP and Cur-SLN at different concentrations. Significance was defined as * *p* < 0.05; **** *p* < 0.0001.

Figure 3 reports the cellular viability of normal OECs after treatment with empty PNP and SLN and with Cur-loaded PNP and SLN. For empty PNP, we found a slight reduction in cell viability compared to the control at all concentrations tested, while we observed a slight increase of viability when cells were treated with Cur-PNP compared to the empty systems. In contrast to PNP, unloaded SLN significantly affected cellular viability compared to the control at all concentrations and this effect is evident even for the cells treated with Cur-SLN, especially at the highest concentration (0.5 and 5 µM).

Overall, empty formulations did not impair cell viability revealing that both nanocarriers did not induce toxic effect and can be used as safe nanocarriers. Cur-PNP and Cur-SLN improved cell proliferation activity compared to the free compound at all concentrations (0.1, 0.5, 5 μM) tested [18]. The difference in cell viability found between Cur-loaded polymeric and lipid NPs can be due to the different composition of these systems.

In the study of Petersan et al., the authors have explored the influence of the type of lipid matrix (trimyristin versus cholesteryl myristate) on cellular viability and IC50 values [53]. They found some correlations between cell viability and formulation composition. In particular, the cholesteryl myristate ester was slightly better tolerated by the cells than the triglyceride trimyristin. Regarding the stabilizing surfactants, different effects were found for stabilizer solutions/dispersions and the respective lipid NP formulations. The most outstanding finding was the strong reduction in cell viability by lipid NPs stabilized with Poloxamer 188. In addition, the effects may, however, be superimposed by those of other parameters like differences in particle size and particle shape [53]. Thus, we can correlate the results of this work suggesting that lipid compared to polymeric materials induced a stronger cellular internalization which could even be attributed to other factors, for example, particle adsorption and particle size; in fact, we observed smaller size for lipid particles compared to the polymeric ones (Table 1).

In order to investigate this phenomenon, viability was also assessed in hypoxic OECs. The in vitro hypoxia condition involves a reduction in the amount of oxygen in the cells, simulating what could happen following neural injury in vivo.

Hypoxia injury is typically involved in the ischemia/reperfusion process, and is closely accompanied with an overload of oxidative stress and mitochondrial dysfunction [54]. Cur acts as an anti-apoptotic enhancer or modifier against oxidative insults, which may integrate its anti-oxidative effect and alterations in signal targets. Even though many studies have shown the appreciable effects of Cur on cell protection, little is known regarding the protective effect of Cur against hypoxia.

**Figure 4:**
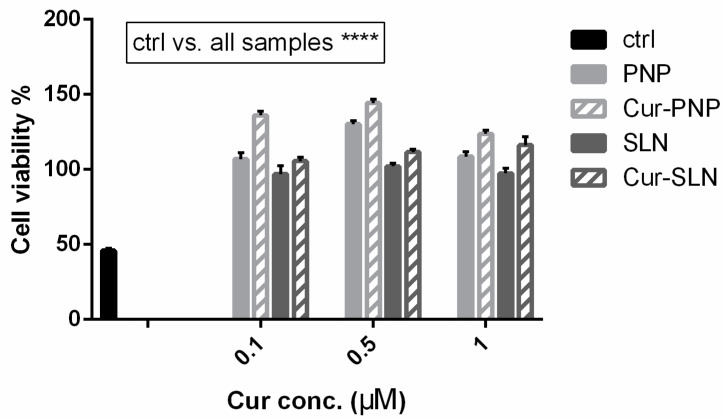
Cell viability after exposure for 24 h of hypoxic OECs to Cur-PNP and Cur-SLN at different concentrations. Significance was defined as **** *p* < 0.0001.

The idea of testing Cur in a hypoxia model stems from the fact that it could counteract the development of reactive oxygen species (ROS). Hypoxia induces the phosphorylation of various signal proteins, such as JNK, ERK, p38 MAPK, caspase-3 and COX-2, increasing the levels of ROS, which play a critical role in neuronal death as occurs in cerebral ischemia. Accordingly, in the study of Ferreira et al., the authors found a dose-dependent neuroprotection provided by immediate and delayed treatment with Cur following neonatal hypoxic-ischemic brain injury. The precise mechanism of this protection is unclear; however, their results showed effects of Cur on oxidative stress and myelination, inflammation and transcription (STAT3 Y705) and mitochondrial dysfunction (STAT3 S727 and PHB) [55]. Considering this, cells cultured under hypoxic conditions can be used as a valid in vitro model in order to evaluate the anti-ROS properties of active molecules such as Cur. Figure 4 reports the effect of Cur-loaded PNP and SLN in hypoxic OECs. In this figure, as well as in Figure 3, the empty nanosuspensions were appropriately diluted to be comparable with the corresponding formulation loaded with the drug. Hypoxia reduces cell proliferation and induces growth arrest, all samples improve cell viability compared to the control and this result is particularly evident with Cur loaded in both nanosuspensions. Surprisingly, in contrast to the normal OECs, we found higher efficacy for both empty and Cur-PNP compared to SLN. Hypoxic conditions alter cell behavior and metabolism, and it could change either the cell membrane structure, composition or both. The alteration of cellular homeostasis could promote PNP permeability and therefore improve their effect compared to healthy cells.

Overall, as mentioned previously, our observations highlight that blank PNP and SLN are nontoxic to OECs. In a previous study, we demonstrated that free Cur increased the viability of hypoxic OECs compared to normal OECs after 6 days of treatment [18]. In contrast, we found that when Cur is loaded into PNP and SLN this effect is evident after 24 h of treatment. Compared to the free drug, both Cur-loaded NPs were more effective against OECs cells over the first 24 h. These findings show that the presence of the nanocarrier system could improve drug internalization into the cells. The results clearly demonstrated that Cur formulated into nanosuspensions was more effective, compared to free drug in terms of cell proliferation, growth and protection. Both the presence of the nanocarriers and the physico-chemical difference between free drug and the nanocarriers systems played a major role in the cellular internalization at all tested drug concentrations. In this regard, SLN and PNP surface composition is a key factor in their cargo internalization pathway and these mechanisms should be analyzed in order to control drug delivery. To explore this hypothesis, uptake studies have been performed on OECs.

### 3.3. Uptake Studies on OECs

Various physicochemical properties of NPs such as surface charge, shape, material composition, surface ligands and surface chemistry are key parameters that determine their intracellular uptake.

The impact of these parameters on cell–NP interactions is very critical because they directly affect the uptake, endocytosis as well as cytotoxicity of NPs [56].

The explanation of cellular uptake results is very complex because in addition to particle properties, before the drug or NPs reach the exterior membranes of target cells, they must interact with the microenvironment around the target cells that can also change the properties of NPs and affect their interactions with the cell membrane and finally their intracellular fate [57].

**Figure 5:**
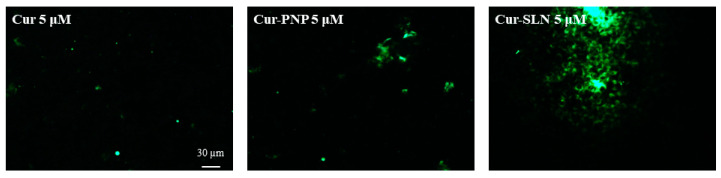
The internalization and uptake of free Cur, Cur-PNP and Cur-SLN into OECs at 5 µM. Scale bar: 30 µm.

As shown in Figure 5, we found a higher internalization for both polymeric and lipid NPs compared to free Cur at the highest drug concentration (5 µM). As reported in Figure S1, Cur concentrations below 5 µM were analyzed but only a very low fluorescence was appreciable, especially with OECs treated with Cur-SLN at 0.5 µM. Probably these concentrations were not detectable by the fluorescent microscope. Moreover, another possible explanation can be the instability of free Cur in contact with cell cultures that could reduce the concentration of drug able to enter into the cells. In fact, degradation of Cur has also been proven after addition to cultured cells. In particular, the stability of Cur in cell culture medium containing 10% FBS or in human blood was improved, but 50% of the compound decomposed after 8 h [58,59].

Accordingly, Schneider et al. found that Cur degrades slower when incubated in the presence of serum or with cultured cells and protein increases the half life of Cur from a few minutes to 1–2 h [60]. When cells have been treated with Cur for several hours the observed effects are due to either Cur, its degradation products or both [60].

The fluorescence signal appears more evident for Cur-SLN at 5 µM (Figure 5), in which drug localization in the cytosolic compartment was visible.

We hypothesized that the different findings observed with Cur-PNP, Cur-SLN and free Cur were due to the physico-chemical property of the drug or particle that is the main factor governing the cellular internalization.

NPs may utilize multiple endocytic pathways depending on their size. Particles with sizes up to 150 nm are mostly internalized via clathrin- or caveolin-mediated endocytosis with a maximum size of 200 nm, while particles ranging from 250 to 3 μm have shown to have an optimal in vitro uptake by macropinocytosis and phagocytosis [61].

Thus, different endocytic pathways correlated with free drug and Cur-loaded PNP and SLN can be supposed. Cur-PNP with dimensions equal to 338 nm could be internalized by macropinocytosis; Cur-SLN with size of 127 nm may be internalized through clathrin- or caveolin-mediated endocytosis while free drug with size >4 µm could be phagocytosed and thus, only low drug levels can permeate into the cells.

Different studies have investigated the relationship between the size of NPs and uptake pathways, but the revealed results have always been inconsistent. These contradictions can be related to the complexity of controlling other parameters of NPs during the process of controlling size; in fact, in most cases a combination of NP parameters synergistically influences a specific biological response.

In addition to that, the size of NPs measured after preparation may undergo changes during the in vitro studies due to agglomeration and aggregation which in turn could affect the cellular internalization pathways [56]. Besides, particle composition may affect the internalization behavior since lipid NPs might enter the cells by passive diffusion, by directly interacting with the lipidic part of the cell membrane (plasma membrane translocation) [61].

Even the shape of NPs plays a pivotal role in the uptake pathway and intracellular trafficking of NPs, as reported in our previous study in which we investigated the uptake of Cur nanocrystals (NCs) in the same cell culture. In particular, the higher internalization of NCs compared to free Cur at the same concentration was probably due to the presence of the stabilizer which acted as penetration enhancer and to the different size and even the shape compared to free Cur. Cur NCs present plate- and needle-like shapes, that could promote their internalization capacity [5].

As previously discussed, Cur-PNP and Cur-SLN present spherical shapes and thus they could be efficiently internalized. Some authors proposed that spherical NPs have a higher cellular uptake rate, other suggested that elongated NPs are better endocytosed than the spherical counterpart [61]. The reasons for these discrepancies are not clear; difference in cellular type may play a significant role in the internalization mechanism. The difference in the uptake performance between Cur-PNP and Cur-SLN both with spherical morphology can be attributed to differences in the surface charge density and surface chemistry of the carriers, the rigidity/flexibility of the particles, the distribution of the nanoparticle size in a sample and difference in the material compositions [62].

In addition to particle uptake by cells, the release of Cur from NPs could promote drug diffusion into the cellular membranes. Therefore, studies have been performed employing several techniques (computational method, i.e., molecular dynamics simulations; electrochemical methods, i.e., cyclic voltammetry and electrochemical impedance spectroscopy) to improve understanding about Cur interaction with cells [63,64]. For the same reason, in our study, DSC analysis has been used to explore Cur (5 µM) behavior with biomembrane models.

### 3.4. In Vitro Biomembrane Model Interactions

The effect of drugs on the structure and function of cell membranes is an important part of the overall effectiveness of a drug. Drug interactions with biological membranes are very complex phenomena. These interactions occur when the biomembrane fences drug passage or when it represents the site of action for the drug. Moreover, drug-membrane interaction can affect the rate of penetration and partitioning of the biomolecule in the cytoplasm to reach a specific target cell organelle or system. Thus, drug partitioning into and binding with cell membranes deserve to be accurately studied and characterized [65]. Although there is a great complexity of biochemical phenomena occurring in living cells, it is possible to exploit a simple experimental model, suitable for investigating or even predicting the possible drug-membrane interactions. Biomembrane models provide the advantage of a system with reduced complexity, even if model membranes will never be able to entirely replace studies using whole cells, they can provide a useful first screening platform for the investigation of drug membrane interactions [66].

Accordingly, liposome can be used as a biomembrane model, the structure of any membrane being a lipid bilayer [67]. Thus, liposome can be developed to mimic the fundamental structural and functional properties of this bilayer being constituted by vesicular phospholipid bilayers that can be relatively easily prepared as unilamellar (ULV) or multilamellar (MLV) structures. The composition of the bilayer can be varied including a wide variety of different lipids and other membrane components. Liposome as model membrane offers an alternative platform to the natural membrane and enables the study of membrane-drug interactions under very defined and controlled conditions.

**Figure 6:**
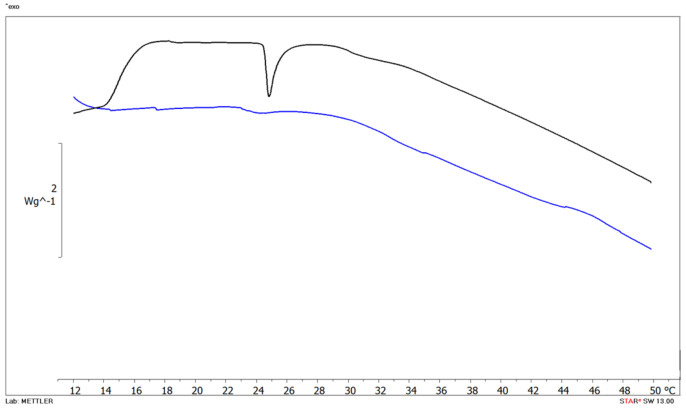
DSC thermograms of empty multilamellar (MLV) structures (black curve) and MLV with Cur 5 µM (blue curve).

The interactions can result in the alteration of either membrane structure, function or both due to the effect of the drug or in a modification in the membrane permeability and fluidity; and conversely, the membrane can affect drug properties, stereochemistry and biological activity. As shown in Figure 6, blank MLV (black curve) showed main phase transition (T_m_) temperature at 24.80 °C corresponding to DMPC transformation from the gel to the liquid crystalline phase (∆H =$\;5.8\times 10^{-5}{\;\mathrm{kJ}\;\mathrm{mol}}^{-1}$).

The amount of Cur added into liposome formulations was selected according to in vitro uptake studies, which revealed some effect at drug concentrations equal to 5 µM. The addition of 5 µM Cur significantly affects DMPC bilayer as evidenced by the disappearance of the T_m._ peak from the calorimetric curves. Cur is highly hydrophobic, and it has indeed been observed partitioning into lipid membranes. The molecule can induce membrane thinning, influence the bilayers’ mechanical properties and change lipid domain behavior.

Two models have been proposed to explain how Cur protects lipid membranes on a molecular level [68]. Some results suggest that Cur lies on the lipid head groups, where it forms hydrogen bonds with the lipid molecules. In this position, Cur can act as a physical barrier, a so-called carpet, preventing peptide or oxidant penetration [68,69]. In contrast, Cur can also embed deeply in the membrane and intercalate with the lipid tails [70,71,72]. Like cholesterol, in this position Cur is proposed to increase lipid chain order and stiffen tails, thereby protecting against peptide insertion. Furthermore, the thermodynamic properties of MLV depend on the molecular structure and composition of the phospholipids. The polar head of phosphatidylcholine is influenced by the length of the hydrocarbon chain, the unsaturations, the asymmetry and branching, the changes in the group that constitutes the polar head and also by the aqueous dispersing medium [73].

In the study by Yumeng Niu et al., the authors found that the encapsulation of Cur in liposomes at highest concentrations (2–20 µM) destroys the structure of the bilayer consisting of a mixture of saturated and unsaturated phospholipids, leading to a more flexible structure [74]. The results obtained can be explained by the planar molecular structure of Cur. In the gel state, the presence of a molecule with these stiffness characteristics can weaken the hydrophobic interactions between the acyl chains of the phospholipids. Therefore, the interaction of Cur with the phospholipid bilayer would lead to a reduction in the ordered structure of the phospholipid chains, increasing the fluidization of the bilayer.

Our data confirm that Cur disorganizes the phospholipid bilayer, as revealed by the peak disappearance at the concentration investigated.

We also found a correlation between thermotropic data and in vitro uptake results. In fact, at the lowest Cur concentrations no fluorescence was detected (Figure 5) and in DSC thermogram (data not shown) no substantial interaction was observed between MLV and the drug; this could probably be explained because the amount of available Cur was below the detection level and the same amount is not able to induce some effect in the biomembrane model. At the highest drug concentrations, a slight fluorescence signal appeared (Figure 5) as well as bilayer perturbation (Figure 6).

Taking into consideration the higher uptake capacity of OECs when Cur was loaded in SLN and PNP, the contact of MLV with Cur-SLN and Cur-PNP should have induced a higher degree of interaction that needs to be investigated. Overall, our results corroborate the scientific literature in which interaction of Cur with biomimetic membrane models have been investigated by using other analytical approaches [63,64].

### 3.5. Stability Studies

In addition to poor solubility, Cur suffers from low chemical stability in aqueous solution and undergoes rapid hydrolysis followed by molecular fragmentation at physiological pH, which has been considered another potential limitation for its therapeutic use. In the study by Wang et al., it was demonstrated that 90% of Cur degraded within 30 min in phosphate buffer at pH 7.4 into trans-6-(40-hydroxy-30-methoxyphenyl)-2,4-dioxo-5-hexenal, ferulic aldehyde, ferulic acid, feruloyl methane and vanillin [58]. Even if already well-known, this phenomenon was also confirmed by our results (Figure S2) which revealed Cur instability in phosphate buffer at pH 5.8 and 7.4 at 25 and 37 °C, respectively. Cur degradation at pH 7.4 (Figure S2A) was correlated to the temperature, as revealed by the residual drug quantity which reached 83% and 54% at 25 and 37 °C, respectively, after 6 h. In contrast, Cur degradation at pH 5.8 (Figure S2B) followed a similar trend, with residual drug quantity of 81 and 73% after 6 h, at 25 and 37 °C, respectively. In accordance with literature, Cur degradation followed first-order kinetics. The degradation rate constants (k) increased with increasing temperature and media pH [75]. These results confirmed that pH is one of the most important factors affecting the stability of a product and that at room temperature degradation was slowed down.

Kharat et al. also found that under acidic conditions, Cur has a tendency to form small crystals that may aggregate when the sample is stirred; while under alkaline conditions, Cur tends to chemically degrade through an autoxidation process [76].

In this regard, nanoencapsulation strategies are necessary to avoid Cur degradation and provide its therapeutic activity. Thus, we aimed to encapsulate Cur and to convert Cur-PNP and Cur-SLN suspensions into a freeze-dried form to bypass drug instability in the aqueous formulation and to investigate the potential stability of the drug. Furthermore, the lyophilization process is crucial to concentrate the formulation that can be resuspended into a small volume as required by the intranasal administration route.

Cur-PNP and Cur-SLN were cryoprotected with glucose being a suitable excipient for both lipid and polymeric nanosuspensions [77,78].

As reported in Table 2, after freeze-drying process Cur-SLN cryoprotected with glucose (5% *w*/*v*), suffered a slight increase in mean size and in PDI value, with no significant variation in ZP values. Cur-PNP maintained almost unaltered mean dimension and ZP values, even if the homogeneity of the formulation was affected compared to the nanosuspension prior to the freeze-drying process. Overall, both formulations were cryoprotected efficiently and maintained suitable parameters in accordance with the administration route under study.

**Figure 7:**
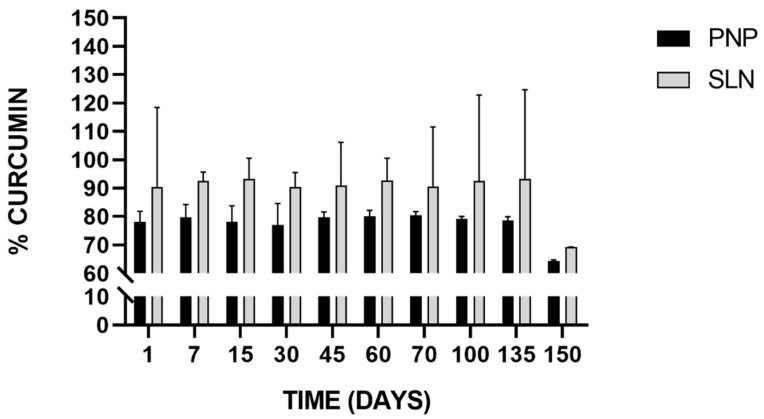
Stability of Cur-loaded PNP and SLN at room temperature up to 150 days.

The ability to remain stable during storage is an important attribute of any colloidal delivery system. The chemical stability of freeze-dried Cur-PNP and Cur-SLN was measured after they were stored at 25 °C for 150 days and then rehydrated in aqueous solution. In Figure 7, the stability results of Cur-loaded PNP and SLN is reported.

Freeze-dried PNP and SLN easily redispersed by shaking and stirring, without additional sonication and homogeneous suspensions were obtained without visible macroscopic particles. After visual appearance, both samples had intensive yellow color; no changes were observed compared to the aqueous suspension prior freeze-drying process.

Particles size, PDI and surface charge remained unchanged during the time intervals considered (data not reported) proving the physical stability of both freeze-dried systems.

SLN successfully preserved drug integrity as revealed by the drug quantity in all samples compared with the initial Cur content. In both cases, the trend can be considered almost constant during the time intervals analyzed except for the last time point, in which we found an appreciable decrease in the Cur percentage in both samples, but more pronounced for SLN which implies that Cur experienced degradation after 150 days. In fact, for up to 135 days, >90% of Cur was retained by SLN stored at room temperature, whereas >70% was retained by PNP stored at the same conditions. These results are encouraging because other authors have investigated Cur stability in nanoparticulate systems but for short-time course (within 1 month) [79,80]. For example, in the study of Fan et al., Cur chemical stability was measured with RP-HPLC at 25 and 95 °C, comparing free Cur and Cur loaded in chitosan NPs and chitosan-chlorogenic acid conjugate NPs. The authors found that Cur retention rate was higher in both NPs compared to free Cur indicating that Cur stability against oxidation or degradation was remarkably improved. Chitosan-chlorogenic acid conjugate showed better chemical stability of Cur at both room temperature and high temperature during 4 h storage [79].

Our findings demonstrated that the incorporation of Cur into both nanosuspensions may protect the drug from degradation at 25 °C for up to 135 days improving its chemical stability, preserving the payload from environmental conditions that may negatively affect it.

## 4. Conclusions

In this study, Cur was efficiently encapsulated in homogeneous PNP and SLN nanosuspensions with suitable properties for potential intranasal administration. Drug encapsulation in both NPs overcomes its chemical instability as revealed by Cur content (>70% and >90% for PNP and SLN, respectively) after 135 days of storage at room temperature as freeze-dried form.

The delivery of the drug through PNP and SLN enhanced OEC viability grown in normal and hypoxic conditions compared with the control and even compared to free Cur after 24 h, indicating the enhanced drug protective effect against injured cells suggesting an increase in Cur antioxidant effect. Cellular uptake studies confirm NPs cellular internalization, showing higher fluorescent signal for Cur-SLN at the highest concentration tested (5 µM), followed by Cur-PNP and free Cur at the same concentrations.

Preliminary interactions study between free drug and the biomembrane model was performed to evaluate potential effects of Cur with biomimetic membrane. Results demonstrated that Cur can perturbate the DMPC bilayer affecting membrane permeability and integrity. Future studies will focus on the effect of Cur-PNP and Cur-SLN with biomembrane models prior to in vivo investigations.

Both formulations can be further investigated as suitable candidates for potential intranasal administration of Cur for neuroprotective effect.

## Figures and Tables

**Table 1:** Physico-chemical characterization and encapsulation efficiency of unloaded and curcumin (Cur)-loaded polymeric nanoparticles (PNP) and lipid nanoparticles (SLN).

Sample	Mean Size ± SD ^a^ (nm)	PDI ^b^ ± SD ^a^	Zeta Potential ± SD ^a^ (mV)	%EE ^c^ ± SD ^a^
PNP	110.00 ± 4.29	0.122 ± 0.089	−20.00 ± 3.50	/
Cur-PNP	338.20 ± 12.25	0.145 ± 0.091	−12.21 ± 0.05	78.04 ± 0.54
SLN	112.90 ± 10.10	0.194 ± 0.03	−32.80 ± 1.20	/
Cur-SLN	127.10 ± 11.30	0.233 ± 0.02	−28.60 ± 1.18	90.49 ± 1.20

^a^ Mean SD, *n* = 3; ^b^ polydispersity index; ^c^ encapsulation efficiency.

**Table 2:** Z-Ave, PDI, and ZP values of Cur-PNP and Cur-SLN before and after the freeze-drying process.

Formulation		Z-Ave ^a^ (nm ± SD ^d^)	PDI ^b^ ± SD ^d^	ZP ^c^ (mV ± SD ^d^)
**Cur-PNP**	Pre-lyophilized	338.20 ±12.25	0.145 ± 0.091	−12.21 ± 0.05
	Post-lyophilized	331.6 ± 0.136	0.527 ± 0.133	−19.7 ± 0.624
**Cur-SLN**	Pre-lyophilized	127.1 ± 11.3	0.233 ± 0.02	−28.6 ± 1.18
	Post-lyophilized	239.0 ± 0.2	0.444 ± 0.2	−24.4 ± 0.1

^a^ Average size; ^b^ Polydispersity index; ^c^ Zeta potential; ^d^ Standard deviation (*n* = 3).

## Data Availability

Data is available on the request from the corresponding author.

## Supplementary Materials

The following are available online at https://www.mdpi.com/2079-4991/11/1/159/s1, Figure S1: The internalization and uptake of free Cur, Cur-PNP and Cur-SLN into OECs at 0.1 and 0.5 µM. Scale bar: 30 µm. Figure S2: Percentage residual drug content for stability determination at pH 7.4 (A) and 5.8 (B) at 25 ± 2 °C and 37 ± 2 °C.
