# Supplementary material for: Curcumin Loaded Polymeric vs. Lipid Nanoparticles: Antioxidant Effect on Normal and Hypoxic Olfactory Ensheathing Cells
Source: Nanomaterials (Basel). 2021 Jan 10;11(1):159. doi: 10.3390/nano11010159 (PMC7827715; doi:10.3390/nano11010159)
Supplement: Supplementary file 1 [file nanomaterials-11-00159-s001.pdf]

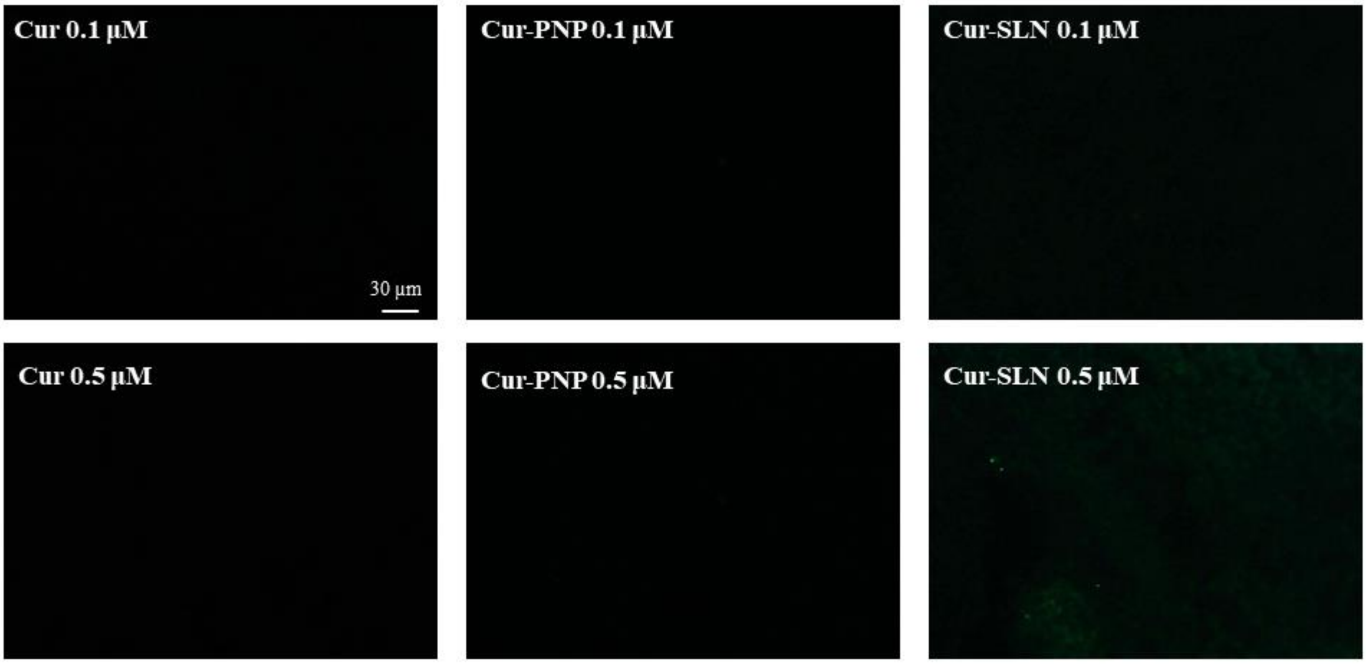


**Figure S1.** The internalization and uptake of free Cur, Cur-PNP and Cur-SLN into OECs at 0.1 and 0.5 µM. Scale bar: 30 µm.

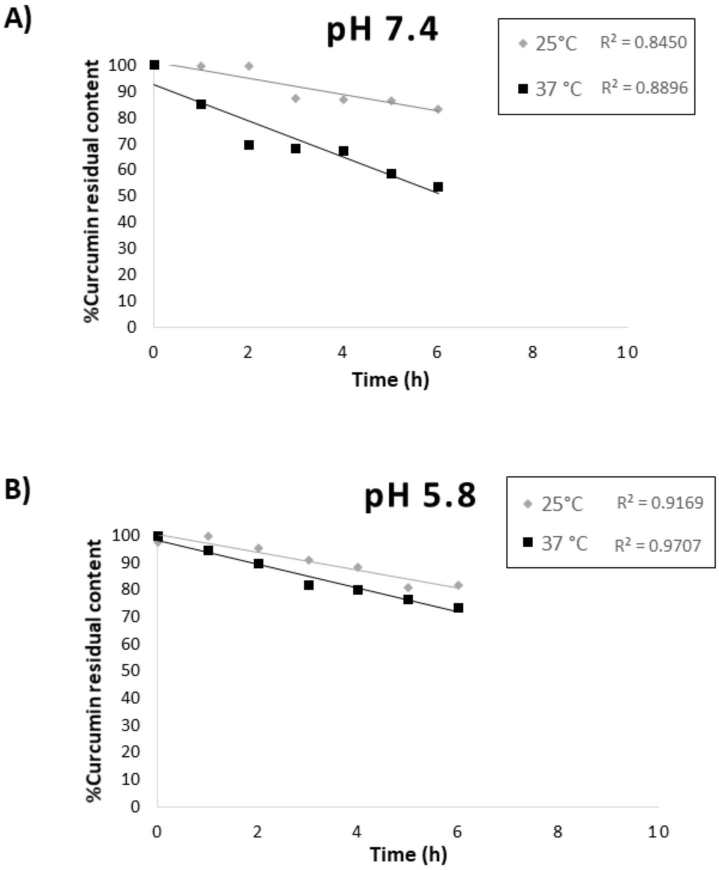


**Figure S2.** Percentage residual drug content for stability determination at pH 7.4 (**A**) and 5.8 (**B**) at 25 ± 2 °C and 37 ± 2 °C.
